# Genomic Surveillance of SARS-CoV-2 Variants in the Dominican Republic and Emergence of a Local Lineage

**DOI:** 10.3390/ijerph20085503

**Published:** 2023-04-13

**Authors:** Robert Paulino-Ramírez, Pablo López, Sayira Mueses, Paula Cuevas, Maridania Jabier, Vanessa Rivera-Amill

**Affiliations:** 1 Instituto de Medicina Tropical y Salud Global, Universidad Iberoamericana, Research Hub, Santo Domingo 22333, Dominican Republic; 2 RCMI Center for Research Resources, Ponce Research Institute, Ponce, PR 00716-2348, USAvrivera@psm.edu (V.R.-A.); 3 Servicio Nacional de Salud (SNS), Ministry of Health, Santo Domingo 10201, Dominican Republic; 4 Basic Sciences Department, School of Medicine, Ponce Health Sciences University, Ponce, PR 00716-2348, USA

**Keywords:** SARS-CoV-2, mutations, variants of concern

## Abstract

Severe acute respiratory syndrome coronavirus 2 (SARS-CoV-2) is an RNA virus that evolves over time, leading to new variants. In the current study, we assessed the genomic epidemiology of SARS-CoV-2 in the Dominican Republic. A total of 1149 SARS-CoV-2 complete genome nucleotide sequences from samples collected between March 2020 and mid-February 2022 in the Dominican Republic were obtained from the Global Initiative on Sharing All Influenza Data (GISAID) database. Phylogenetic relationships and evolution rates were analyzed using the maximum likelihood method and the Bayesian Markov chain Monte Carlo (MCMC) approach. The genotyping details (lineages) were obtained using the Pangolin web application. In addition, the web tools Coronapp, and Genome Detective Viral Tools, among others, were used to monitor epidemiological characteristics. Our results show that the most frequent non-synonymous mutation over the study period was D614G. Of the 1149 samples, 870 (75.74%) were classified into 8 relevant variants according to Pangolin/Scorpio. The first Variants Being Monitored (VBM) were detected in December 2020. Meanwhile, in 2021, the variants of concern Delta and Omicron were identified. The mean mutation rate was estimated to be 1.5523 × 10^−3^ (95% HPD: 1.2358 × 10^−3^, 1.8635 × 10^−3^) nucleotide substitutions per site. We also report the emergence of an autochthonous SARS-CoV-2 lineage, B.1.575.2, that circulated from October 2021 to January 2022, in co-circulation with the variants of concern Delta and Omicron. The impact of B.1.575.2 in the Dominican Republic was minimal, but it then expanded rapidly in Spain. A better understanding of viral evolution and genomic surveillance data will help to inform strategies to mitigate the impact on public health.

## 1. Introduction

Severe acute respiratory syndrome coronavirus 2 (SARS-CoV-2), responsible for an unprecedented global health crisis, has caused more than 6 million deaths since it was first detected in China in late 2019 [1]. SARS-CoV-2 is a single-stranded, positive-sense RNA virus belonging to the Coronaviridae family, with an approximately 30,000-base-pair genome [2]. This recent betacoronavirus belongs to a family of zoonotic viruses with sylvatic animal reservoirs and a complex history of spillovers in the last 20 years [3]. The beginning of the coronavirus disease (COVID) pandemic was marked by two major lineages: A and B [4]. Among these, lineage B has been more widely spread throughout the pandemic, including the reference genome Wuhan/Hu-1/2019 [5].

Reconstructing viral ancestral haplotypes has been part of the scientific framework since the identification of SARS in 2002 [6]. Several studies propose separate events associated with the SARS-CoV-2 origins that today serve as a template for the wild-type sequence [7,8,9]. Between March and May of 2020, a rapidly spreading variant bearing a glycine residue at position 614 in the spike protein, replacing an aspartic acid residue (D614G or G614), with increased transmissibility, was associated with the first wave in Western countries [10]. SARS-CoV-1 and SARS-CoV-2 share almost 79% of sequence identity and use the angiotensin-converting enzyme 2 (ACE2) receptor for cell entry. The S protein is one of the virus’s most critical regions as it mediates the interaction with human cells [10]. The binding of the virus with ACE2 on the membrane host cells facilitates viral entry, a crucial determinant of infectivity and pathogenesis [11,12]. Continuous receptor binding domain (RBD) mutations on the spike protein are primarily associated with its rapid evolutionary turnovers facilitating viral fitness and immunological resistance [13,14].

The Dominican Republic is a democratic nation in the Greater Caribbean, sharing the island of La Hispaniola with the Republic of Haiti. The first confirmed cases in the Dominican Republic were reported in late February 2020 among travelers from Italy, predominantly the B.1 variant carrying the D614G mutation [15]. As of March 2020, more than 500,000 cases were confirmed, and the country experienced five consecutive waves. The Dominican Republic’s initial response focused on eliminating transmission chains with a national emergency declaration from 19 March 2020 until mid-2021 [16]. In addition, collective actions were structured around *Quedate en Casa* policies (stay-at-home), the closure of schools, the economy, borders, and increased testing, tracing, and public and private laboratory capacity for the diagnosis of symptomatic and asymptomatic individuals. Epidemiological data collected during the first years of the pandemic on the island suggest that confirmed cases and deaths associated with COVID-19 fluctuated according to government restrictions and non-pharmaceutical interventions [17,18].

Despite all efforts, a serological study conducted between April and June 2020 in emerging hotspots revealed that anti-SARS-CoV-2 IgM and IgG positivities were 3.8% and 5.4%, respectively, indicating a rapid expansion and cryptic community transmission. According to the 2019 Global Health Security (GHS) Index, the DR was underprepared for the early detection and molecular surveillance of emerging and re-emerging pathogens compared with other Latin American countries [19,20]. However, we established a scientific partnership between local health authorities, academia, and international centers to deploy a genomic surveillance initiative to further analyze complete SARS-CoV-2 genomes to ease the understanding of variant and lineage circulation in multiple provinces and municipalities in the Dominican Republic [15,21]. We performed a comprehensive analysis of SARS-CoV-2 genome sequences for sequences available from March 2020 until February 2022. Our study aimed to analyze SARS-CoV-2 genomic and phylogenetic data from the Dominican Republic, characterize introduction events, and assess autochthonous lineage circulation.

## 2. Materials and Methods

### 2.1. Ethics Statement

The current study was conducted in accordance with the Declaration of Helsinki, and the protocol was certified by the Institutional Review Board of the Ponce Research Institute to be exempt from the federal policy for the protection of human subjects under the provision of the use of existing data (IRB protocol number 2203092896; approval date 10 July 2022).

### 2.2. Epidemiological Data

We used the number of COVID-19 confirmed cases reported by the Dominican Republic Department of Health from March 2020 to February 2022 using 91-DIVOC (https://91-divoc.com/pages/covid-visualization/ (accessed on 24 May 2022)). The collection includes cases classified as confirmed and plotted by date of sample collection.

### 2.3. SARS-CoV-2 Genome Sequences and Nucleotide Aligment

A total of 1149 SARS-CoV-2 whole-genome nucleotide sequences from the Dominican Republic were obtained from the Global Initiative on Sharing All Influenza Data (GISAID) database [22,23,24]. Of these, 940 sequences included the entire spike gene region and were selected to perform the analyses. The sequences used to assess the course of the viral epidemic over time were collected between March 2020 and mid-February 2022 in the Dominican Republic. The sequences are around 3819 base pairs long, starting at position 21,563 and ending at position 25,381, relative to the reference sequence NC_045512.2 [5,25,26]. Nucleotide sequences were input in FASTA format into the Multiple Alignment Program for Amino Acid or Nucleotide Sequences (MAFFT version 7) to perform the alignment of sequences [27]. The final alignments were performed using BioEdit Sequence Alignment Editor (v 7.2.5) [28]. To collapse the set of sequences and reduce redundancy, we used Jalview (v 2.11.1.5) [29]. All genome sequences used in this study are published in the GISAID database with the following identifiers: GISAID Identifier EPI_SET_230302bt doi: 10.55876/gis8.230302bt; EPI_SET_230302kx doi 10.55876/gis8.230302kx; EPI_SET_230302qv doi: 10.55876/gis8.230302qv.

### 2.4. Phylogenetic Analyses

Phylogenetic relationships were analyzed using the maximum likelihood method implemented in the IQ tree web server with a re-sampling process (branch support analysis) with 1000 bootstraps [30]. The obtained consensus tree was then visualized using the Interactive Tree Of Life (iTOL) available at https://itol.embl.de/ (accessed on 25 March 2022) [31]. The evolution rate (nucleotide substitutions, site, year) of SARS-CoV-2 in the Dominican Republic during 2020, 2021, and early 2022 was evaluated using the Bayesian Markov chain Monte Carlo (MCMC) approach implemented in BEAST (v1.10.4) [32]. Data were first imported to BEAUti, which is part of the BEAST software package, and dates were parsed according to the collection date. Exponential growth and relaxed molecular clock were used as a coalescent tree before measuring the evolutionary change over time [33]. The general time-reversible model (GTR) suggested by Modeltest was used to perform the analysis with gamma/invariant sites as a site heterogeneity model [34]. Markov chains were generated after 200,000,000 generations, with sampling carried out every 10,000 generations. To summarize the posterior distribution, we created a consensus tree discarding the first 10% as burn-in using TreeAnnotator software (part of the BEAST software package). Tracer software version 1.7.1 was used to produce the demographic reconstruction and to calculate the effective sample size (ESS) [35]. A Bayesian skyline plot (BSP) was generated to estimate the demographic history from the evaluated sequences through time by using coalescent inference approaches. The obtained plots represent the relative genetic diversity estimated using the effective number of infections (y = Ne) through time (x = t). The solid line in the plot represents the median estimated effective population size, with the 95% highest and lowest posterior densities (credibility interval) in gray [36].

### 2.5. SARS-CoV-2 Lineage Assingment and S Gene Mutations

The genotyping details (lineages) were obtained using the Pangolin web application (v. 3.1.16), which is a computational tool that has been developed to assign lineages to COVID-19 sequences based on the methodology described by O’Toole et al. [37]. The viral variant designations were confirmed using the Serious Constellations of Reoccurring Phylogenetically-independent Origin (Scorpio) application [38]. The web tools Coronapp and Genome Detective Viral Tools were used to monitor mutations [25,39].

### 2.6. Detection of SARS-CoV-2 Autochthonous Transmission

To track and analyze the variation in SARS-CoV-2 sequences from the Dominican Republic, sequences were input into the CoV-GLUE online web application [40,41]. We also used CoV-GLUE to identify autochthonous SARS-CoV-2 transmission [40,41].

### 2.7. Data Availability

SARS-CoV-2 genomes with GISAID accession numbers used in this study are available in Supplementary Table S1. To view the contributors of each individual sequence with details such as the accession number, virus name, collection date, originating lab and submitting lab, and list of authors, please visit 10.55876/gis8.230302bt; 10.55876/gis8.230302kx; 10.55876/gis8.230302qv. All sequences in this dataset are compared relative to hCoV-19/Wuhan/WIV04/2019 (WIV04), the official reference sequence employed by GISAID (EPI_ISL_402124).

## 3. Results

### 3.1. Characterization of SARS-CoV-2 Variants Circulating in the Dominican Republic from March 2020 to February 2022

**Figure 1:**
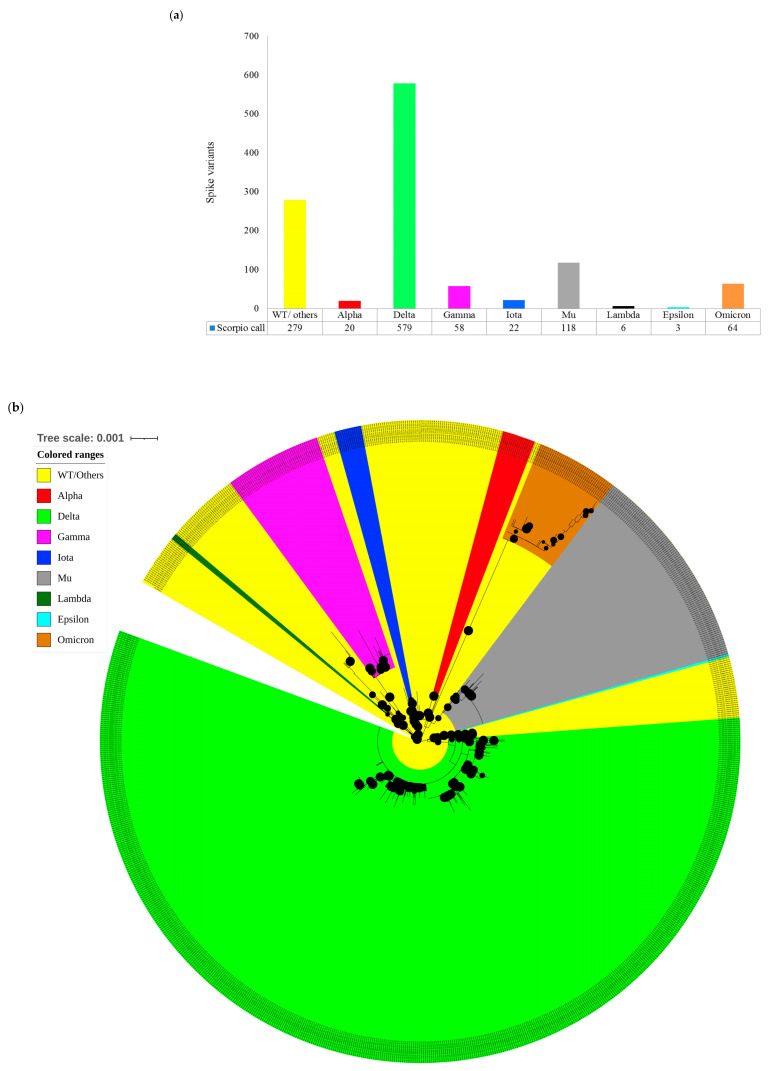
Detection and characterization of SARS-CoV-2 variants circulating in the Dominican Republic from March 2020 to February 2022. (**a**) The SARS-CoV-2 variant designations were obtained using the Pango lineage website (https://cov-lineages.org/ (accessed on 25 February 2022)) (n = 1149). Lineages not confirmed by Scorpio were grouped into WT or others. (**b**) The phylogenetic evaluation was inferred using the maximum likelihood method implemented in the IQ TREE web server. The bootstrap consensus tree was inferred from 1000 replicates. The colors indicate the SARS-CoV-2 spike variant, and the bullets (•) indicate branches with a bootstrap value of >0.70. The visualization was created using the online software tool iTOL v.5 (**b**).

We conducted genomic surveillance for twenty-three months starting from March 2020. The sequences used in our study were from samples collected in the Dominican Republic between March 2020 and mid-February 2022. Of the 1149 sequences, 870 (75.7%) were grouped into 8 relevant variants according to Pangolin/Scorpio. The maximum likelihood tree indicated that Omicron is distinct from the other variants observed (Figure 1).

**Figure 2:**
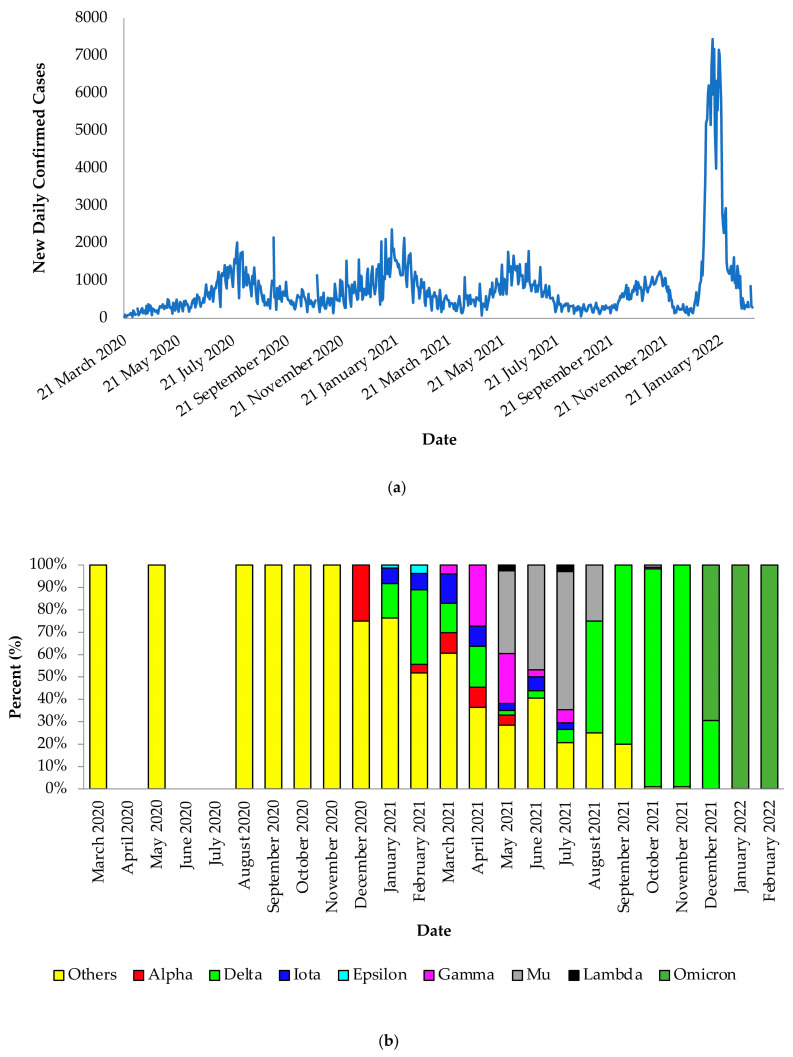
Daily SARS-CoV-2 confirmed cases and variant distribution between March 2020 and February 2022. (**a**) The daily reported trends were obtained using 91-DIVOC (https://91-divoc.com/pages/covid-visualization/ (accessed on 5 May 2022). (**b**) The full genome nucleotide sequences and their genotyping details were obtained from the Global Initiative on Sharing All Influenza Data (GISAID) database and Pangolin web application, respectively.

From March 2020 to February 2022, we observed five epidemic waves with high points in August 2020, January 2021, June 2021, November 2021, and January 2022 (Figure 2a). Our data include SARS-CoV-2 genomes from the beginning of the epidemic transmission in the Dominican Republic in March 2020. According to our data, wild-type viruses and viruses not listed as under monitoring or of concern were observed in 2020, except for Alpha, first detected in December 2020. In early 2021, the variants Alpha, Delta, Iota, Epsilon, and Gamma were identified. While there was co-circulation of SARS-CoV-2 variants during the first half of 2021, the Delta variant of concern became the predominant variant by August 2021. Towards the end of December 2021, the variant of concern Omicron was first detected and continued to be the dominant variant throughout January and February 2022 (Figure 2b, Supplementary Figure S1).

### 3.2. Monitoring Mutations and Estimating the Mutation Rate of SARS-CoV-2 Spike Protein from Sequences Obtained in the Dominican Republic

**Figure 3:**
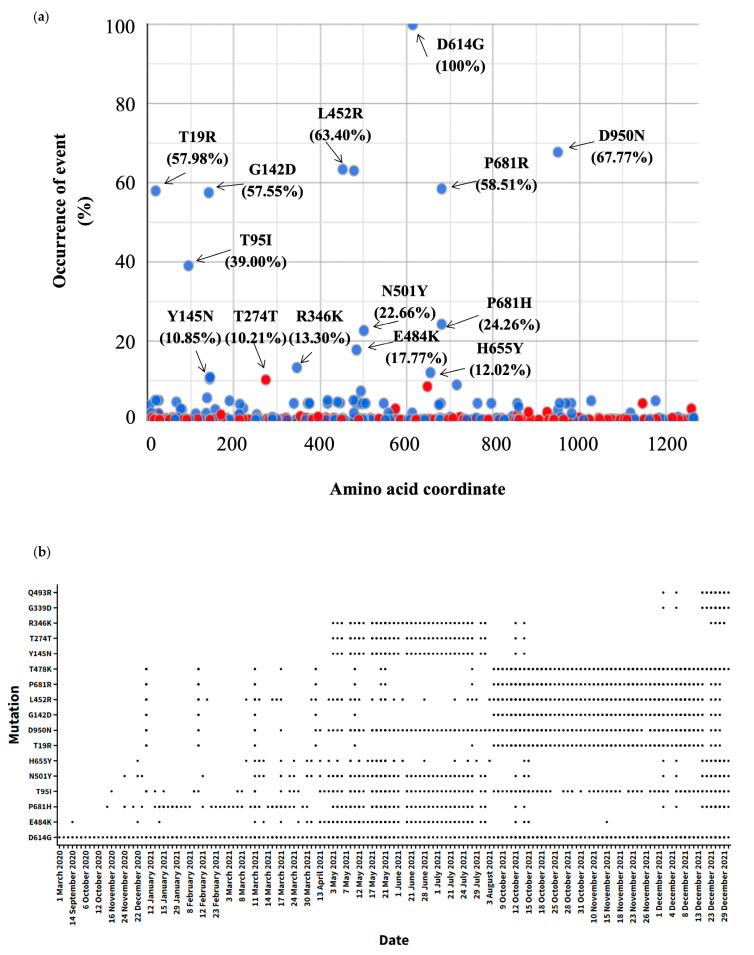
Monitoring and identification of SARS-CoV-2 spike mutations. (**a**) Three hundred and ninety-three different mutations were detected in the sequences (n = 940) used in the evaluation. The webtool coronapp was used to determine the mutations (mutation frequency for S protein (spike) in percent). Blue and red colors indicate amino acid changes and silent mutations, respectively. The arrows indicate the mutation. (**b**) Distribution of the most relevant mutations in the spike protein (X > 10%) and two key Omicron mutations (G339D, Q493R) through time. The black spots represent the detection of the mutation through time.

We also analyzed the mutations in the spike protein through time. After evaluating sequence redundancies in the spike protein, 393 mutations at the amino acid level were detected in the sequences (n = 940) used in the evaluation (Figure 3a). Our results showed that the number of mutations in the S protein increased over time (Figure 3b). The most frequent non-synonymous mutation during the study period was D614G (100%). In addition to D614G, 14 amino acid mutations were observed with a high frequency (X > 10%), among which D950N (67.77%), L452R (63.40%), T478K (63.09%), D681R (58.51%), T19R (57.98%), and T95I (39.00%) were the most abundant. By October 2021, the mutations P681H (24.26%), N501Y (22.66%), and H655Y (12.02), after their initial identification in late 2020 among the circulating variants, were no longer detected, but they re-emerged in December 2021 during the Omicron wave, along with the key mutations G339D and Q493R (Figure 3). The T474K, P681R, L452R, G142D, and T19R mutations were identified in early 2021. However, the identification of all these mutations decreased in mid-2021. However, it increased again in August 2021, and these mutations were detected throughout the rest of the evaluation period. The mutations R346K, T274T, Y145N, and E484N were identified with a high frequency in mid-2021 (May to October). Meanwhile, the E484K mutation was detected as early as September 2020, but it was no longer detected after November 2021.

**Figure 4:**
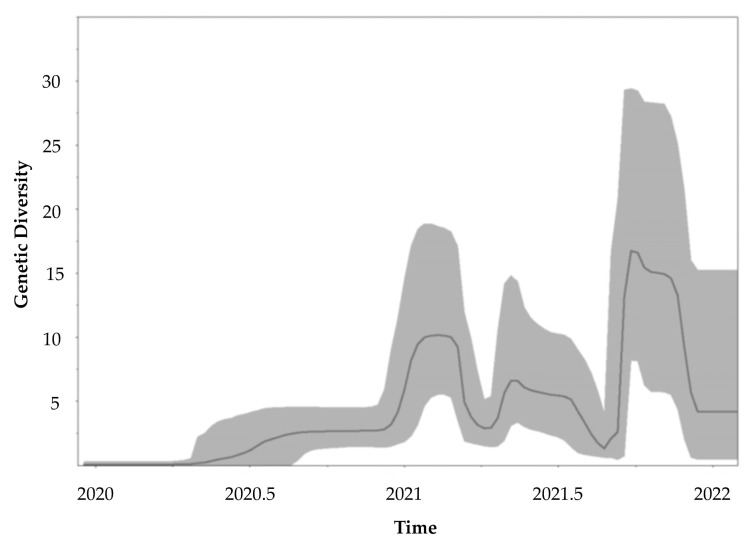
Molecular tracing of SARS-CoV-2 in the Dominican Republic. The evolution rate was evaluated by using the Bayesian Markov chain Monte Carlo (MCMC) approach implemented in BEAST (v1.10.4). Tracer software v 1.7.1 was used to produce the demographic reconstruction. The Y-axis represents the relative genetic diversity estimated using the effective number of infections through time on the X-axis. The solid line represents the median value, and the gray area represents the 95% credibility intervals.

The mean mutation rate was estimated to be 1.5523 × 10^−3^ (95% HPD: 1.2358 × 10^−3^, 1.8635 × 10^−3^) nucleotide substitutions per site. Figure 4 shows the Bayesian skyline plot describing the SARS-CoV-2 viral evolution dynamics. The demographic reconstruction shows an initial short lag phase, after which the epidemic experienced a fast exponential growth from early 2021, followed by an asymptotic phase towards the end of 2021 (Figure 4).

### 3.3. Emergence of Autochthonous Lineage B.1.575.2

**Figure 5:**
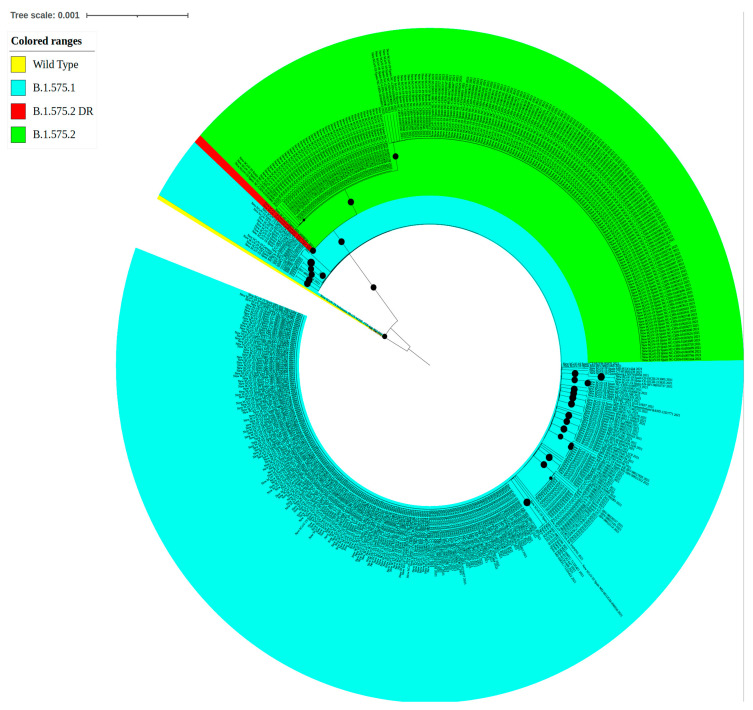
Maximum likelihood phylogenetic analysis of the B.1.575 lineage. The phylogenetic evaluation of the B.1.575 lineage was inferred using the maximum likelihood method implemented in the IQ TREE web server. The bootstrap consensus tree was inferred from 1000 replicates. The colors indicate the SARS-CoV-2 spike variant, and the bullets (•) indicate branches with a bootstrap value of >0.70. The visualization was created using the online software tool iTOL v.5 (B).

Our analysis also showed the evolution and emergence of a SARS-CoV-2 variant in the Dominican Republic. Interpretations of the CoV-GLUE online platform suggest that the SARS-CoV-2 sublineage B.1.575.2 emerged in the Dominican Republic (https://cov-glue.cvr.gla.ac.uk/lineage.php?lineage=B.1.575.2 (accessed on 11 October 2022)) (Supplementary Figure S2). We detected lineage B.1.575.2 in October 2021, during the fourth epidemic wave (Figure 2a). Lineage B.1.575.2 co-circulated with the variants of concern Delta and Omicron (Figure 5). The B.1.575.2 lineage did not become a predominant lineage in the Dominican Republic (Figure 2B). The latest date of detection of B.1.575.2 corresponded to a sample from January 2022. Following this initial period, the SARS-CoV-2 lineage B.1.575.2 was no longer detected in the Dominican Republic, but it expanded rapidly in Spain [42].

## 4. Discussion

Upon the first reported cases of COVID-19, the Dominican Republic government implemented a strict lockdown driven by the lack of vaccines and effective non-pharmaceutical interventions, with a gradual relaxation of the measures starting in August 2020 with the reactivation of tourism and other economic activities [43,44,45]. However, despite local health authorities’ initial containment measures for COVID-19 cases in early 2020, we experienced a rapid increase in the transmission of the virus throughout the island, facilitated by travel and continuous intra-island transmission. The Dominican Republic has a well-developed tourism transportation network with constant and heavy trafficking of people between the Caribbean countries, North America, and some European countries. The continuous travel could explain why two SARS-CoV-2 lineages emerged in the Dominican Republic and expanded to other countries such as Spain, the United States, and Puerto Rico. In the Dominican Republic, like in many parts of the world, it is evident that several phases of viral evolution occurred, and we aimed to describe the molecular epidemiology of SARS-CoV-2 transmission in the Dominican Republic.

The S protein is the main target of the current vaccines [11]. Therefore, the high prevalence of non-synonymous substitution events in this protein, which can alter the functional properties of the resulting protein, has a particular significance in the evolution of SARS-CoV-2 [12]. In SARS-CoV-2 sequences from the Dominican Republic, we observed a gradual accumulation of mutations during the initial period of the second year of the pandemic, with new variants emerging and spreading rapidly. The mutation rate of the S protein in the Dominican Republic (1.5523 × 10^−3^ (95% HPD: 1.2358 × 10^−3^, 1.8635 × 10^−3^) is consistent with previous studies [46,47,48,49]. The first year (2020) of the pandemic in the Dominican Republic was characterized by low sequence diversity with an initial phase where the spike protein mutation D614G became dominant and was maintained during the rest of the study period. This fast exponential growth is consistent with the accumulation of mutations and emergence of new variants. The population size, the high rate of virion replication within the host, the prolonged duration of the pandemic, and the continuous population dynamics, among other factors, contributed to the expansion of multiple variants [50].

The emergence of new variants is often associated with high transmission levels, a higher proportion of unvaccinated individuals, prolonged transmission periods, and, in some cases, the result of selective pressures such as immune response or antiviral use. However, to our knowledge, at the end of 2020, no official regimens of specific antivirals were available in the Dominican Republic. In December 2020, Public Health England (PHE) announced a new variant, B.1.1.7 (Alpha), with a significant increase in transmission [51]. While the mutations in this variant increased viral infectivity (N501Y, E484K, P681H, D614G, and the deletion H69V70—Δ69-70), they had no apparent role in antibody evasion mechanisms [52,53]. The Alpha variant rapidly spread worldwide, and as we report here, the Dominican Republic reported its first case in December 2020, lasting until mid-2021. The Alpha variant was replaced in the Dominican Republic by the Mu (B.1.621), Iota (B.1.526), Gamma (P1), and Delta (B.1.617.2) variants. In contrast, the Epsilon (B.1.4727/429) variant was detected in early 2021, where it spread slowly and was replaced within two months. In October 2021, we detected the emergence of B.1.575.2, a sublineage of B.1.575 that emerged in the United States (https://cov-glue.cvr.gla.ac.uk/lineage.php?lineage=B.1.575 (accessed on 11 October 2022), and after its emergence, two new sublineages were identified: B.1.575.1 (first reported in Colombia), and B.1.575.2 (first detected in the Dominican Republic). The Delta variant was reported in October 2020 in India and identified in other countries such as the UK in late 2020, which rapidly spread among the population [54]. Our analysis indicates that the Delta variant was present as early as January 2021 and persisted until the end of the year. During the last months of 2021, this variant became the most dominant in the Dominican Republic due to its increased replication adaptability and the decreased sensitivity to neutralizing antibodies [55].

The emergence and circulation of the B.1.575.2 variant in the Dominican Republic during the latter half of 2021 might not be associated with a change in the community transmission variables. This is partly because of the co-circulation of other variants with higher transmissibility rates, such as B.1.1.7 (Alpha) introduced and registered in the country after reopening aerial and terrestrial borders and the reactivation of tourism. However, this variant was later detected in Spain [42], where it caused an increase in the transmission and hospitalization parameters. While the impact of this variant was minimal in the Dominican Republic, some intrinsic factors may have influenced the short molecular detection period, including (a) the place of emergence within small remote communities with a low to very low demographic distribution, or (b) its circulation within areas of limited surveillance.

The World Health Organization (WHO) reported the emergence of Omicron (B.1.1.529) in November 2021. The variant was first detected in Botswana and was rapidly designated as a variant of concern due to the rapid increase in cases in South Africa [56]. The most important characteristics were an unprecedented number of previously described and novel mutations and their spread worldwide within a short period [57]. Omicron harbors up to 30 mutations throughout the spike protein compared to approximately 10 substitutions in both the SARS-CoV-2 Alpha and Delta variants of concern, and it spreads at a previously unseen rate [58,59]. The high number of mutations raised much concern because this region mediates host entry and is the main target of neutralizing antibodies [57]. The most representative mutations include A67V, Δ69-70, T95I, G142D, Δ143-145, Δ211/L212I, ins214EPE, G339D, S371L, S373P, S375F, K417N, N440K, G446S, S477N, T478K, E484A, Q493R, G496S, Q498R, N501Y, Y505H, T547K, D614G, H655Y, N679K, P681H, N764K, D796Y, N856K, Q954H, N969K, and L981F, some of which are associated with increases in transmissibility, vaccine resistance, and the risk of reinfection [59,60]. Some of these mutations are present in variants that circulated previously and may lead to similar detrimental outcomes in the host [59].

Currently, the link between the variant of concern Omicron with its predecessors remains unclear. However, three possible explanations have been proposed: inadequate genomic surveillance in the region where it originated, prolonged viral shedding in chronically infected people, and an alternative zoonotic origin [61,62,63,64]. Interestingly, the most common symptoms observed in other variants tend to be less severe in this variant, therefore reducing the risk of hospitalization among symptomatic cases [65,66,67]. In early December 2021, the first case of Omicron was reported in the Dominican Republic, and in late December, the number of cases increased rapidly. The Omicron variant and subvariants have been demonstrated to be more immunologically evasive and environmentally persistent than the ancestral strains. The high rate of mutation and increased infectivity will be challenging for surveillance systems worldwide [68,69,70,71,72,73,74].

The major limitation of this study was the lack of epidemiological information. In addition, we only evaluated 81.81% of the Dominican Republic sequences available in the GISAID database in mid-February 2022. However, evaluating phylogenetic inferences from a large-scale dataset is challenging due to the increased computational demand [75]. Additional data should help to define the evolutionary history of the virus.

## 5. Conclusions

This study provides a synopsis of the genomic epidemiology of SARS-CoV-2 in the Dominican Republic. SARS-CoV-2 is an RNA virus that is characterized by the accumulation of changes over time and the production of new variants. Each new emerging variant of concern is more infectious than the preceding variant it displaced. Continuous evolution has occurred in almost all regions of the SARS-CoV-2 genome and potentially in a country-specific manner. Our study provides evidence of a SARS-CoV-2 lineage, B.1.575.2, initially registered in the Dominican Republic without an epidemiological impact, which later provoked increased transmission in Spain. The documentation of local autochthonous lineage transmission is crucial for implementing public health containment measures as the emergence of highly adapted SARS-CoV-2 variants may lead to future pandemics. The agenda to prevent future pandemics must include strategies for the timely identification of emerging or previously circulating pathogens, including assessments in the most distant communities and in places where there are no operational reporting parameters. For several reasons, low-income countries or countries with poor satellite laboratory systems would face the most significant implementation challenges. However, efforts can be brought together at the regional level and in collaboration through health diplomacy.

We still have many elements to understand about the highly adaptive capacity of SARS-CoV-2, its recombinant forms, and its ability to remain in transmission networks without rapidly raising epidemiological alerts. To avoid the emergence and re-emergence of zoonotic viruses, we must strengthen the first pillar of pandemic preparation: genomic surveillance.

## Data Availability

A list of all the sequences used in this study can be found in Supplementary Table S1.

## Supplementary Materials

The following supporting information can be downloaded at: https://www.mdpi.com/article/10.3390/ijerph20085503/s1. Figure S1. Scorpio call SARS-CoV-2 variant assignment over time. Data represents 186 different data collection date and 1149 sequences. Figure S2. Pangolin lineage os SARS-CoV-2 variant assignment over time. Data represents 186 different data collection date and 1149 sequences. Table S1. Accession numbers of the sequences used in the study. All genome sequences used in this study are published in GISAID’s data base with the following identifiers: GISAID Identifier: EPI_SET_230302bt doi: 10.55876/gis8.230302bt; EPI_SET_230302kx doi: 10.55876/gis8.230302kx; EPI_SET_230302qv doi: 10.55876/gis8.230302qv.
