# Supplementary material for: Genomic Surveillance of SARS-CoV-2 Variants in the Dominican Republic and Emergence of a Local Lineage
Source: Int J Environ Res Public Health. 2023 Apr 13;20(8):5503. doi: 10.3390/ijerph20085503 (PMC10138544; doi:10.3390/ijerph20085503)
Supplement: Supplementary file 1 [file ijerph-20-05503-s001.zip › ijerph-2140002-supplementary.pdf]

Figure S1. Scorpio call SARS-CoV-2 variant assignment over time. Data represents 186 different data collection date and 1,149 sequences.

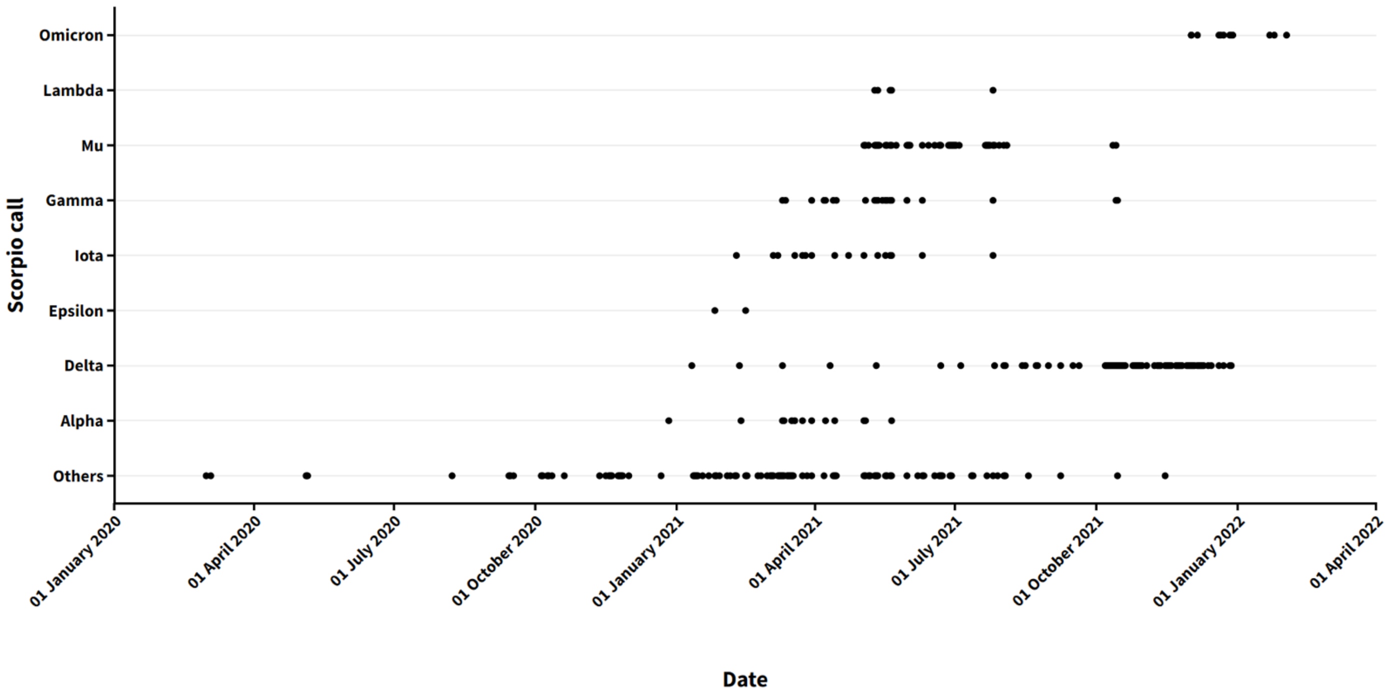

Figure S2. Pangolin lineage of SARS-CoV-2 variant assignment over time. Data represents 186 different data collection date and 1,149 sequences.

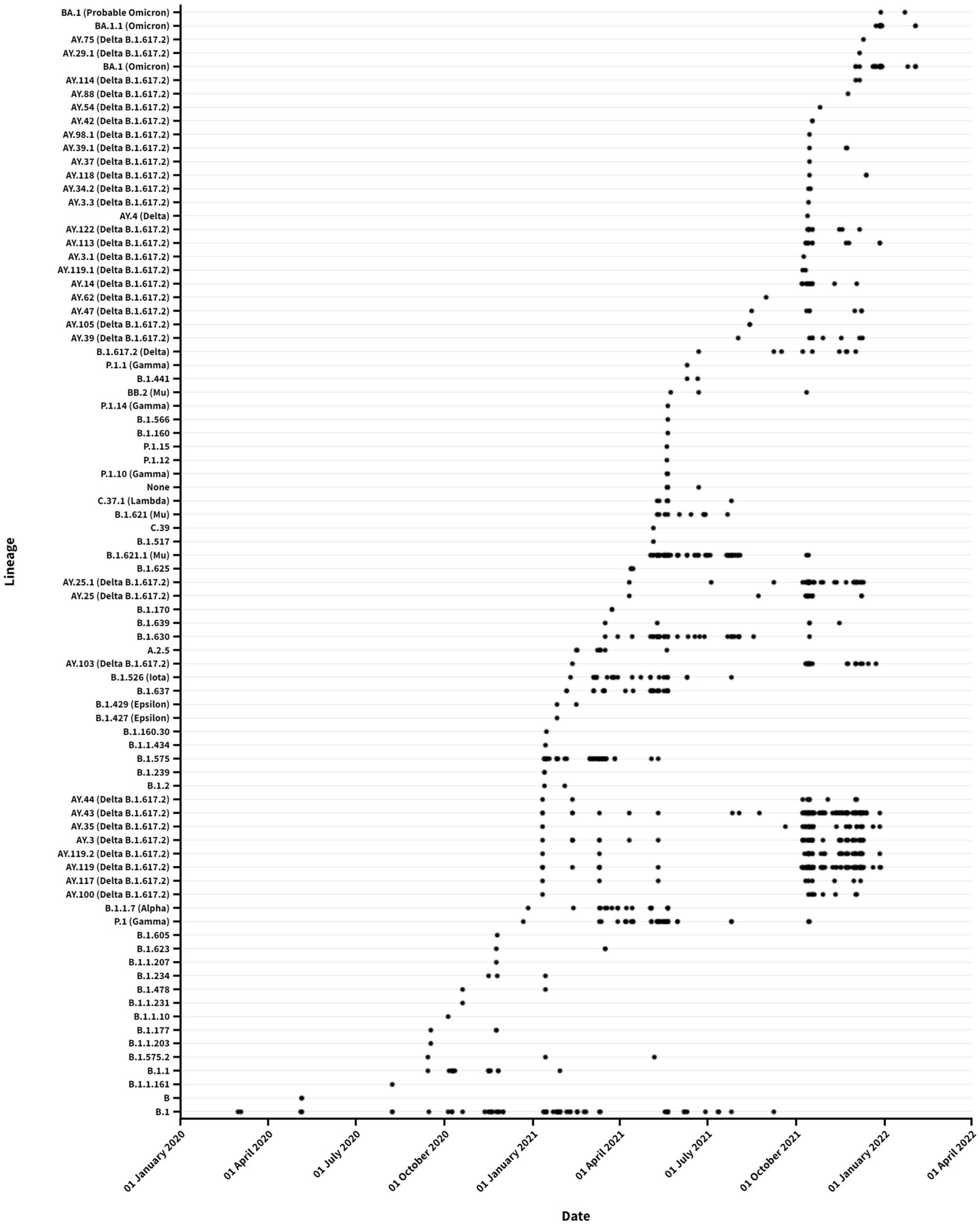

Table S1. Accession numbers of the sequences used in the study. All genome sequences used in this study are published in GISAID's data base with the following identifiers: GISAID Identifier: EPI_SET_230302bt doi: 10.55876/gis8.230302bt; EPI_SET_230302kx doi 10.55876/gis8.230302kx; EPI_SET_230302qv doi: 10.55876/gis8.230302qv.

Accession ID	Sample Collection Date
EPI_ISL_6897916	10/7/21
EPI_ISL_6897924	10/8/21
EPI_ISL_6897928	10/8/21
EPI_ISL_6897936	10/8/21
EPI_ISL_6897944	10/8/21
EPI_ISL_6897949	10/9/21
EPI_ISL_6897952	10/11/21
EPI_ISL_6897959	10/11/21
EPI_ISL_6897962	10/11/21
EPI_ISL_6897963	10/11/21
EPI_ISL_6897970	10/11/21
EPI_ISL_6897977	10/11/21
EPI_ISL_6897987	10/11/21
EPI_ISL_6897993	10/12/21
EPI_ISL_6898000	10/12/21
EPI_ISL_6898002	10/12/21
EPI_ISL_6898004	10/12/21
EPI_ISL_6898012	10/12/21
EPI_ISL_6898017	10/12/21
EPI_ISL_6898031	10/13/21
EPI_ISL_6898032	10/13/21
EPI_ISL_6898040	10/13/21
EPI_ISL_6898049	10/13/21
EPI_ISL_6898052	10/13/21
EPI_ISL_6898053	10/13/21
EPI_ISL_6898066	10/13/21
EPI_ISL_6898076	10/13/21
EPI_ISL_6898077	10/14/21
EPI_ISL_6898083	10/14/21
EPI_ISL_6898091	10/14/21
EPI_ISL_6898099	10/14/21
EPI_ISL_6898100	10/14/21
EPI_ISL_6898107	10/14/21

Accession ID	Sample Collection Date
EPI_ISL_6898111	10/14/21
EPI_ISL_6898117	10/14/21
EPI_ISL_6898123	10/14/21
EPI_ISL_6898124	10/14/21
EPI_ISL_6898128	10/14/21
EPI_ISL_6898134	10/14/21
EPI_ISL_6898138	10/14/21
EPI_ISL_6898139	10/14/21
EPI_ISL_6898146	10/14/21
EPI_ISL_6898151	10/14/21
EPI_ISL_6898158	10/14/21
EPI_ISL_6898166	10/14/21
EPI_ISL_6898167	10/14/21
EPI_ISL_6898173	10/14/21
EPI_ISL_6898179	10/14/21
EPI_ISL_6898185	10/14/21
EPI_ISL_6898186	10/14/21
EPI_ISL_6898201	10/15/21
EPI_ISL_6898209	10/15/21
EPI_ISL_6898210	10/15/21
EPI_ISL_6898219	10/15/21
EPI_ISL_6898220	10/15/21
EPI_ISL_6898223	10/15/21
EPI_ISL_6898224	10/15/21
EPI_ISL_6898230	10/15/21
EPI_ISL_6898237	10/15/21
EPI_ISL_6898243	10/15/21
EPI_ISL_6898244	10/15/21
EPI_ISL_6898249	10/15/21
EPI_ISL_6898259	10/15/21
EPI_ISL_6898260	10/16/21
EPI_ISL_6898264	10/17/21
EPI_ISL_6898273	10/17/21
EPI_ISL_6898280	10/17/21
EPI_ISL_6898286	10/17/21
EPI_ISL_6898293	10/18/21
EPI_ISL_6898294	10/18/21
EPI_ISL_6898299	10/18/21
EPI_ISL_6898301	10/18/21

Accession ID	Sample Collection Date
EPI_ISL_6898302	10/18/21
EPI_ISL_6898304	10/18/21
EPI_ISL_6898311	10/18/21
EPI_ISL_6898315	10/18/21
EPI_ISL_6898325	10/18/21
EPI_ISL_6898326	10/18/21
EPI_ISL_6898336	10/18/21
EPI_ISL_6898341	10/19/21
EPI_ISL_6898342	10/19/21
EPI_ISL_6898947	10/14/21
EPI_ISL_6898948	10/13/21
EPI_ISL_6898950	10/11/21
EPI_ISL_6898963	10/12/21
EPI_ISL_6898964	10/15/21
EPI_ISL_6898965	10/8/21
EPI_ISL_6898970	10/8/21
EPI_ISL_6898971	10/9/21
EPI_ISL_6898976	10/13/21
EPI_ISL_6898977	10/14/21
EPI_ISL_6898978	10/15/21
EPI_ISL_6898979	10/18/21
EPI_ISL_6898991	10/15/21
EPI_ISL_6899009	10/16/21
EPI_ISL_6899017	10/15/21
EPI_ISL_6899018	10/14/21
EPI_ISL_6899025	10/13/21
EPI_ISL_6899054	10/14/21
EPI_ISL_6899062	10/14/21
EPI_ISL_6899070	10/13/21
EPI_ISL_6899071	10/15/21
EPI_ISL_6899076	10/19/21
EPI_ISL_6899086	10/15/21
EPI_ISL_6897948	10/8/21
EPI_ISL_6897981	10/11/21
EPI_ISL_6898023	10/13/21
EPI_ISL_6898060	10/13/21
EPI_ISL_6898097	10/14/21
EPI_ISL_525468	5/6/20
EPI_ISL_6898157	10/14/21

**Accession ID**	**Sample Collection Date**
EPI_ISL_6898192	10/15/21
EPI_ISL_6898320	10/18/21
EPI_ISL_6898353	10/20/21
EPI_ISL_6898348	10/19/21
EPI_ISL_6898949	10/11/21
EPI_ISL_6898954	10/18/21
EPI_ISL_6898990	10/14/21
EPI_ISL_6899026	10/14/21
EPI_ISL_6899089	10/18/21
EPI_ISL_2501664	1/13/21
EPI_ISL_2501665	1/15/21
EPI_ISL_2501670	1/15/21
EPI_ISL_2501671	1/13/21
EPI_ISL_2501698	1/26/21
EPI_ISL_2501700	1/15/21
EPI_ISL_2501701	2/5/21
EPI_ISL_2501702	1/15/21
EPI_ISL_2501707	1/14/21
EPI_ISL_2501708	1/13/21
EPI_ISL_2501709	2/9/21
EPI_ISL_2501710	1/15/21
EPI_ISL_2501711	1/15/21
EPI_ISL_2501712	1/15/21
EPI_ISL_2501713	1/18/21
EPI_ISL_2501714	1/26/21
EPI_ISL_2501715	1/15/21
EPI_ISL_2501716	1/15/21
EPI_ISL_2501717	1/13/21
EPI_ISL_2501720	2/5/21
EPI_ISL_2501723	2/5/21
EPI_ISL_2501728	1/14/21
EPI_ISL_2501729	1/13/21
EPI_ISL_2501730	1/13/21
EPI_ISL_3827558	6/10/21
EPI_ISL_2501718	2/3/21
EPI_ISL_906851	12/27/20
EPI_ISL_2776156	3/19/21
EPI_ISL_2776157	3/19/21
EPI_ISL_2557422	3/1/21

**Accession ID**	**Sample Collection Date**
EPI_ISL_2557423	3/27/21
EPI_ISL_2557424	3/11/21
EPI_ISL_6368762	5/21/21
EPI_ISL_6368767	5/10/21
EPI_ISL_6368777	5/10/21
EPI_ISL_6368782	5/18/21
EPI_ISL_6368785	5/10/21
EPI_ISL_6368792	5/21/21
EPI_ISL_6368797	5/10/21
EPI_ISL_6368803	5/21/21
EPI_ISL_6368807	5/21/21
EPI_ISL_6368808	5/21/21
EPI_ISL_6368809	5/10/21
EPI_ISL_6368810	5/10/21
EPI_ISL_6368812	5/20/21
EPI_ISL_6368813	5/21/21
EPI_ISL_6368814	5/20/21
EPI_ISL_6368815	5/20/21
EPI_ISL_6368816	5/10/21
EPI_ISL_6368817	5/20/21
EPI_ISL_6368818	5/17/21
EPI_ISL_6368819	5/20/21
EPI_ISL_6368820	5/15/21
EPI_ISL_6368821	5/21/21
EPI_ISL_6368822	5/20/21
EPI_ISL_6368823	5/17/21
EPI_ISL_6368824	5/18/21
EPI_ISL_6368825	5/17/21
EPI_ISL_6368826	5/10/21
EPI_ISL_6368827	5/20/21
EPI_ISL_6368828	5/17/21
EPI_ISL_6368829	5/17/21
EPI_ISL_6368830	5/21/21
EPI_ISL_6368831	5/10/21
EPI_ISL_6368832	5/10/21
EPI_ISL_6368833	5/21/21
EPI_ISL_6368834	5/21/21
EPI_ISL_6368835	5/10/21
EPI_ISL_6368836	5/10/21

Accession ID	Sample Collection Date
EPI_ISL_6368837	5/18/21
EPI_ISL_6368838	5/21/21
EPI_ISL_6368839	5/21/21
EPI_ISL_6368840	5/21/21
EPI_ISL_6368841	5/21/21
EPI_ISL_6368842	5/21/21
EPI_ISL_6368843	5/21/21
EPI_ISL_6368844	5/10/21
EPI_ISL_6368845	5/10/21
EPI_ISL_6368846	5/17/21
EPI_ISL_6368847	5/10/21
EPI_ISL_6368848	5/13/21
EPI_ISL_6368849	5/17/21
EPI_ISL_6368850	5/10/21
EPI_ISL_6368851	5/17/21
EPI_ISL_6368852	5/10/21
EPI_ISL_6368853	5/20/21
EPI_ISL_6368854	5/17/21
EPI_ISL_6368855	5/17/21
EPI_ISL_6368856	5/17/21
EPI_ISL_6368857	5/17/21
EPI_ISL_6368858	5/21/21
EPI_ISL_6368859	5/24/21
EPI_ISL_6368860	5/21/21
EPI_ISL_6368861	5/17/21
EPI_ISL_6368862	5/10/21
EPI_ISL_6368863	5/20/21
EPI_ISL_6368864	5/21/21
EPI_ISL_6368869	5/21/21
EPI_ISL_6368870	5/17/21
EPI_ISL_6368871	5/10/21
EPI_ISL_6368872	5/20/21
EPI_ISL_6368873	5/20/21
EPI_ISL_2776158	3/24/21
EPI_ISL_2776159	3/26/21
EPI_ISL_2776161	3/5/21
EPI_ISL_2776164	3/30/21
EPI_ISL_2776165	3/30/21
EPI_ISL_2776166	3/30/21

Accession ID	Sample Collection Date
EPI_ISL_2776169	3/8/21
EPI_ISL_2776170	3/8/21
EPI_ISL_2776171	4/15/21
EPI_ISL_2776172	4/15/21
EPI_ISL_2776173	4/15/21
EPI_ISL_2776174	4/13/21
EPI_ISL_2776176	4/14/21
EPI_ISL_2776177	4/23/21
EPI_ISL_2776178	4/7/21
EPI_ISL_2776181	4/8/21
EPI_ISL_2776184	1/14/21
EPI_ISL_2776185	1/14/21
EPI_ISL_2776188	1/14/21
EPI_ISL_2776193	1/26/21
EPI_ISL_2776195	1/26/21
EPI_ISL_2776196	1/26/21
EPI_ISL_2776197	1/26/21
EPI_ISL_2776198	1/26/21
EPI_ISL_2776201	1/26/21
EPI_ISL_2776202	1/26/21
EPI_ISL_2776203	1/27/21
EPI_ISL_2776206	4/14/21
EPI_ISL_4220360	7/26/21
EPI_ISL_2273999	3/1/21
EPI_ISL_2274001	3/4/21
EPI_ISL_2274002	3/10/21
EPI_ISL_2274003	3/10/21
EPI_ISL_2274005	3/13/21
EPI_ISL_2274006	3/15/21
EPI_ISL_2274007	3/15/21
EPI_ISL_2274008	3/11/21
EPI_ISL_2274009	3/12/21
EPI_ISL_2274010	3/12/21
EPI_ISL_2274011	3/9/21
EPI_ISL_2274012	3/15/21
EPI_ISL_2274013	3/15/21
EPI_ISL_2274014	3/16/21
EPI_ISL_2274015	3/16/21
EPI_ISL_2274016	3/15/21

**Accession ID**	**Sample Collection Date**
EPI_ISL_2274017	3/15/21
EPI_ISL_2274018	3/17/21
EPI_ISL_2274019	3/16/21
EPI_ISL_2274020	3/17/21
EPI_ISL_2274021	3/17/21
EPI_ISL_2274022	3/17/21
EPI_ISL_2274023	3/17/21
EPI_ISL_2274024	3/17/21
EPI_ISL_3104700	5/12/21
EPI_ISL_3105426	1/26/21
EPI_ISL_3105436	4/13/21
EPI_ISL_3105537	1/22/21
EPI_ISL_3827499	6/10/21
EPI_ISL_3827767	6/10/21
EPI_ISL_3828021	3/15/21
EPI_ISL_4220348	7/22/21
EPI_ISL_4220349	7/22/21
EPI_ISL_4220351	8/14/21
EPI_ISL_4220352	7/22/21
EPI_ISL_4220353	7/21/21
EPI_ISL_4220354	7/22/21
EPI_ISL_4220355	8/4/21
EPI_ISL_4220356	7/26/21
EPI_ISL_4220357	7/26/21
EPI_ISL_4220358	7/26/21
EPI_ISL_4220359	8/2/21
EPI_ISL_4220361	7/30/21
EPI_ISL_4220362	7/29/21
EPI_ISL_4220363	8/2/21
EPI_ISL_4220364	8/2/21
EPI_ISL_4220365	7/27/21
EPI_ISL_4220366	7/26/21
EPI_ISL_4220367	8/3/21
EPI_ISL_4220368	7/26/21
EPI_ISL_4220369	8/2/21
EPI_ISL_4220370	8/3/21
EPI_ISL_4220371	7/26/21
EPI_ISL_4220372	7/24/21
EPI_ISL_4220373	7/27/21

**Accession ID**	**Sample Collection Date**
EPI_ISL_4220374	7/26/21
EPI_ISL_4220375	7/26/21
EPI_ISL_4220376	7/26/21
EPI_ISL_4220377	7/23/21
EPI_ISL_4220378	7/23/21
EPI_ISL_4220379	8/2/21
EPI_ISL_4220380	7/26/21
EPI_ISL_4220381	8/3/21
EPI_ISL_4220382	7/26/21
EPI_ISL_4220383	7/27/21
EPI_ISL_4220384	8/14/21
EPI_ISL_2776194	1/26/21
EPI_ISL_2776167	3/5/21
EPI_ISL_2776160	3/5/21
EPI_ISL_2776162	3/5/21
EPI_ISL_2776163	3/30/21
EPI_ISL_2776168	3/5/21
EPI_ISL_2776175	4/14/21
EPI_ISL_2776179	4/7/21
EPI_ISL_2776180	4/7/21
EPI_ISL_2776182	4/8/21
EPI_ISL_2776183	1/14/21
EPI_ISL_2776186	1/14/21
EPI_ISL_2776187	1/14/21
EPI_ISL_2776189	1/14/21
EPI_ISL_2776190	1/14/21
EPI_ISL_2776191	1/14/21
EPI_ISL_2776192	1/26/21
EPI_ISL_2776199	1/26/21
EPI_ISL_2776200	1/26/21
EPI_ISL_2776204	1/27/21
EPI_ISL_2776205	1/13/21
EPI_ISL_4220350	7/26/21
EPI_ISL_3045367	3/12/21
EPI_ISL_3045368	3/15/21
EPI_ISL_3045369	3/16/21
EPI_ISL_3045385	5/21/21
EPI_ISL_3045388	5/21/21
EPI_ISL_3045389	5/18/21

**Accession ID**	**Sample Collection Date**
EPI_ISL_3045390	5/18/21
EPI_ISL_3045391	5/21/21
EPI_ISL_3045392	5/21/21
EPI_ISL_3045395	5/21/21
EPI_ISL_3045398	5/21/21
EPI_ISL_3045401	5/20/21
EPI_ISL_3045402	5/20/21
EPI_ISL_3045405	5/21/21
EPI_ISL_3045406	5/21/21
EPI_ISL_3045407	5/21/21
EPI_ISL_3045408	5/21/21
EPI_ISL_3045409	5/21/21
EPI_ISL_3045410	5/21/21
EPI_ISL_3045411	5/21/21
EPI_ISL_3045412	5/21/21
EPI_ISL_3045413	5/21/21
EPI_ISL_3045416	5/21/21
EPI_ISL_3045417	5/21/21
EPI_ISL_3045418	5/21/21
EPI_ISL_3045419	5/20/21
EPI_ISL_3045421	5/20/21
EPI_ISL_3104659	5/10/21
EPI_ISL_3104663	5/4/21
EPI_ISL_3104664	5/6/21
EPI_ISL_3104666	5/12/21
EPI_ISL_3104668	5/10/21
EPI_ISL_3104669	5/3/21
EPI_ISL_3104670	5/6/21
EPI_ISL_3104671	5/10/21
EPI_ISL_3104672	5/12/21
EPI_ISL_3104673	5/12/21
EPI_ISL_3104674	5/12/21
EPI_ISL_3104678	5/11/21
EPI_ISL_3104680	5/12/21
EPI_ISL_3104683	5/4/21
EPI_ISL_3104684	5/4/21
EPI_ISL_3104685	5/3/21
EPI_ISL_3104690	5/3/21
EPI_ISL_3104691	5/6/21

**Accession ID**	**Sample Collection Date**
EPI_ISL_3104695	5/4/21
EPI_ISL_3104696	5/4/21
EPI_ISL_3104697	5/10/21
EPI_ISL_3104698	5/10/21
EPI_ISL_3104699	5/12/21
EPI_ISL_3104701	5/12/21
EPI_ISL_3104702	5/18/21
EPI_ISL_3104706	5/10/21
EPI_ISL_3104707	5/12/21
EPI_ISL_3104708	5/12/21
EPI_ISL_3104710	5/12/21
EPI_ISL_3104717	5/11/21
EPI_ISL_3104718	5/12/21
EPI_ISL_3104719	5/6/21
EPI_ISL_3104722	5/7/21
EPI_ISL_3104723	5/11/21
EPI_ISL_3104724	5/12/21
EPI_ISL_3104753	5/3/21
EPI_ISL_3104754	5/4/21
EPI_ISL_3104755	5/4/21
EPI_ISL_3104756	5/4/21
EPI_ISL_3104757	5/4/21
EPI_ISL_3104758	5/6/21
EPI_ISL_3104759	5/6/21
EPI_ISL_3104760	5/6/21
EPI_ISL_3104761	5/11/21
EPI_ISL_3104762	5/11/21
EPI_ISL_3104763	5/12/21
EPI_ISL_3104794	5/3/21
EPI_ISL_3104795	5/3/21
EPI_ISL_3105428	4/13/21
EPI_ISL_3105435	2/9/21
EPI_ISL_3105438	1/27/21
EPI_ISL_3105439	1/14/21
EPI_ISL_3105444	1/26/21
EPI_ISL_3105450	3/24/21
EPI_ISL_3105467	1/26/21
EPI_ISL_3105468	3/26/21
EPI_ISL_3105488	3/30/21

Accession ID	Sample Collection Date
EPI_ISL_3105491	1/12/21
EPI_ISL_3105501	4/15/21
EPI_ISL_3105502	4/13/21
EPI_ISL_3105503	4/13/21
EPI_ISL_3105535	3/24/21
EPI_ISL_3105536	1/15/21
EPI_ISL_3105538	1/13/21
EPI_ISL_3105539	3/24/21
EPI_ISL_3105544	1/26/21
EPI_ISL_6437538	3/17/21
EPI_ISL_6437539	3/12/21
EPI_ISL_6437540	3/16/21
EPI_ISL_6437541	3/17/21
EPI_ISL_6437542	3/16/21
EPI_ISL_6437543	3/12/21
EPI_ISL_6437544	3/16/21
EPI_ISL_6437545	3/16/21
EPI_ISL_6437546	3/5/21
EPI_ISL_6437547	3/8/21
EPI_ISL_6437548	3/8/21
EPI_ISL_6437549	3/5/21
EPI_ISL_6437550	3/16/21
EPI_ISL_6437551	3/15/21
EPI_ISL_6437552	3/17/21
EPI_ISL_6437553	3/3/21
EPI_ISL_6437554	3/14/21
EPI_ISL_6437555	3/17/21
EPI_ISL_3188565	5/12/21
EPI_ISL_3188570	5/12/21
EPI_ISL_3188571	5/12/21
EPI_ISL_3188572	5/10/21
EPI_ISL_3188598	5/4/21
EPI_ISL_3188599	5/6/21
EPI_ISL_3188600	5/6/21
EPI_ISL_3188603	5/10/21
EPI_ISL_3188604	5/10/21
EPI_ISL_3188605	5/11/21
EPI_ISL_3188606	5/12/21
EPI_ISL_3188607	5/12/21

Accession ID	Sample Collection Date
EPI_ISL_3188617	5/10/21
EPI_ISL_3236433	5/18/21
EPI_ISL_3236434	5/18/21
EPI_ISL_3236435	5/18/21
EPI_ISL_3236436	5/18/21
EPI_ISL_3236437	5/18/21
EPI_ISL_3236438	5/18/21
EPI_ISL_3236439	5/18/21
EPI_ISL_3236440	5/31/21
EPI_ISL_3236441	5/31/21
EPI_ISL_3236442	5/31/21
EPI_ISL_3236443	5/31/21
EPI_ISL_3236444	5/31/21
EPI_ISL_3236445	6/1/21
EPI_ISL_3236446	6/2/21
EPI_ISL_3236447	6/10/21
EPI_ISL_3236448	6/14/21
EPI_ISL_3236449	6/29/21
EPI_ISL_3236450	6/29/21
EPI_ISL_3236451	6/29/21
EPI_ISL_3236452	6/11/21
EPI_ISL_8482323	10/8/21
EPI_ISL_8482325	10/8/21
EPI_ISL_8482334	10/11/21
EPI_ISL_8482324	10/8/21
EPI_ISL_8482326	10/9/21
EPI_ISL_8482316	10/7/21
EPI_ISL_8482327	10/9/21
EPI_ISL_8482330	10/11/21
EPI_ISL_8482317	10/8/21
EPI_ISL_8482328	10/9/21
EPI_ISL_8482318	10/8/21
EPI_ISL_8482329	10/10/21
EPI_ISL_8482319	10/8/21
EPI_ISL_8482320	10/8/21
EPI_ISL_8482331	10/11/21
EPI_ISL_8482321	10/8/21
EPI_ISL_8482332	10/11/21
EPI_ISL_8482322	10/8/21

Accession ID	Sample Collection Date
EPI_ISL_8482333	10/11/21
EPI_ISL_8482346	10/12/21
EPI_ISL_8482345	10/12/21
EPI_ISL_8482335	10/11/21
EPI_ISL_8482336	10/11/21
EPI_ISL_8482347	10/12/21
EPI_ISL_8482337	10/11/21
EPI_ISL_8482349	10/12/21
EPI_ISL_8482348	10/12/21
EPI_ISL_8482338	10/11/21
EPI_ISL_8482339	10/11/21
EPI_ISL_8482341	10/12/21
EPI_ISL_8482350	10/12/21
EPI_ISL_8482340	10/11/21
EPI_ISL_8482351	10/13/21
EPI_ISL_8482342	10/12/21
EPI_ISL_8482343	10/12/21
EPI_ISL_8482344	10/12/21
EPI_ISL_8482356	10/13/21
EPI_ISL_8482357	10/13/21
EPI_ISL_8482358	10/14/21
EPI_ISL_8482360	10/14/21
EPI_ISL_8482359	10/14/21
EPI_ISL_8482361	10/14/21
EPI_ISL_8482362	10/14/21
EPI_ISL_8482352	10/13/21
EPI_ISL_8482353	10/13/21
EPI_ISL_8482354	10/13/21
EPI_ISL_8482355	10/13/21
EPI_ISL_8482370	10/14/21
EPI_ISL_8482367	10/14/21
EPI_ISL_8482378	10/15/21
EPI_ISL_8482363	10/14/21
EPI_ISL_8482374	10/14/21
EPI_ISL_8482368	10/14/21
EPI_ISL_8482369	10/14/21
EPI_ISL_8482371	10/14/21
EPI_ISL_8482372	10/14/21
EPI_ISL_8482373	10/14/21

**Accession ID**	**Sample Collection Date**
EPI_ISL_8482375	10/15/21
EPI_ISL_8482365	10/14/21
EPI_ISL_8482376	10/15/21
EPI_ISL_8482366	10/14/21
EPI_ISL_8482377	10/15/21
EPI_ISL_8482389	10/15/21
EPI_ISL_8482388	10/15/21
EPI_ISL_8482379	10/15/21
EPI_ISL_8482393	10/15/21
EPI_ISL_8482381	10/15/21
EPI_ISL_8482392	10/15/21
EPI_ISL_8482382	10/15/21
EPI_ISL_8482383	10/15/21
EPI_ISL_8482394	10/15/21
EPI_ISL_8482384	10/15/21
EPI_ISL_8482385	10/15/21
EPI_ISL_8482386	10/15/21
EPI_ISL_8482387	10/15/21
EPI_ISL_8482390	10/15/21
EPI_ISL_8482380	10/15/21
EPI_ISL_8482391	10/15/21
EPI_ISL_8482395	10/15/21
EPI_ISL_8482396	10/15/21
EPI_ISL_8482397	10/15/21
EPI_ISL_8482398	10/15/21
EPI_ISL_8482399	10/17/21
EPI_ISL_8482364	10/14/21
EPI_ISL_8482796	10/18/21
EPI_ISL_8482797	10/18/21
EPI_ISL_8482787	10/15/21
EPI_ISL_8482790	10/15/21
EPI_ISL_8482798	10/18/21
EPI_ISL_8482786	10/15/21
EPI_ISL_8482799	10/18/21
EPI_ISL_8482785	10/15/21
EPI_ISL_8482788	10/15/21
EPI_ISL_8482789	10/15/21
EPI_ISL_8482791	10/15/21
EPI_ISL_8482792	10/16/21

**Accession ID**	**Sample Collection Date**
EPI_ISL_8482793	10/16/21
EPI_ISL_8482794	10/17/21
EPI_ISL_8482795	10/17/21
EPI_ISL_8482780	10/13/21
EPI_ISL_8482781	10/13/21
EPI_ISL_8482782	10/13/21
EPI_ISL_8482783	10/14/21
EPI_ISL_8482784	10/14/21
EPI_ISL_8482837	10/20/21
EPI_ISL_8482834	10/19/21
EPI_ISL_8482830	10/19/21
EPI_ISL_8482831	10/19/21
EPI_ISL_8482832	10/19/21
EPI_ISL_8482833	10/19/21
EPI_ISL_8482835	10/19/21
EPI_ISL_8482836	10/20/21
EPI_ISL_8482826	10/19/21
EPI_ISL_8482827	10/19/21
EPI_ISL_8482828	10/19/21
EPI_ISL_8482829	10/19/21
EPI_ISL_8482820	10/18/21
EPI_ISL_8482821	10/18/21
EPI_ISL_8482823	10/18/21
EPI_ISL_8482822	10/18/21
EPI_ISL_8482824	10/18/21
EPI_ISL_8482825	10/18/21
EPI_ISL_8482815	10/18/21
EPI_ISL_8482816	10/18/21
EPI_ISL_8482817	10/18/21
EPI_ISL_8482818	10/18/21
EPI_ISL_8482819	10/18/21
EPI_ISL_8482810	10/18/21
EPI_ISL_8482811	10/18/21
EPI_ISL_8482813	10/18/21
EPI_ISL_8482812	10/18/21
EPI_ISL_8482814	10/18/21
EPI_ISL_8482804	10/18/21
EPI_ISL_8482805	10/18/21
EPI_ISL_8482806	10/18/21

Accession ID	Sample Collection Date
EPI_ISL_8482803	10/18/21
EPI_ISL_8482808	10/18/21
EPI_ISL_8482807	10/18/21
EPI_ISL_8482809	10/18/21
EPI_ISL_8482800	10/18/21
EPI_ISL_8482801	10/18/21
EPI_ISL_8482802	10/18/21
EPI_ISL_5587613	6/18/21
EPI_ISL_5587621	6/21/21
EPI_ISL_5587633	6/21/21
EPI_ISL_5587645	6/22/21
EPI_ISL_5587651	6/22/21
EPI_ISL_5587660	6/22/21
EPI_ISL_5587664	6/22/21
EPI_ISL_5587676	9/20/21
EPI_ISL_5587688	9/8/21
EPI_ISL_5587695	9/8/21
EPI_ISL_5587703	8/24/21
EPI_ISL_5621195	6/27/21
EPI_ISL_5621494	6/28/21
EPI_ISL_5621499	6/30/21
EPI_ISL_5621502	7/1/21
EPI_ISL_9405828	11/8/21
EPI_ISL_9405831	11/10/21
EPI_ISL_9405836	11/11/21
EPI_ISL_9405837	11/12/21
EPI_ISL_9405838	11/12/21
EPI_ISL_9405839	11/11/21
EPI_ISL_9405842	11/8/21
EPI_ISL_9405864	11/18/21
EPI_ISL_9405845	11/12/21
EPI_ISL_9405848	11/15/21
EPI_ISL_9405846	11/15/21
EPI_ISL_9405847	11/15/21
EPI_ISL_9405849	11/15/21
EPI_ISL_9405857	11/17/21
EPI_ISL_9405851	11/15/21
EPI_ISL_9405856	11/15/21
EPI_ISL_9405852	11/16/21

**Accession ID**	**Sample Collection Date**
EPI_ISL_9405853	11/16/21
EPI_ISL_9405855	11/16/21
EPI_ISL_9405858	11/17/21
EPI_ISL_9405860	11/16/21
EPI_ISL_9405865	11/18/21
EPI_ISL_9405874	11/22/21
EPI_ISL_9405869	11/22/21
EPI_ISL_9405879	11/22/21
EPI_ISL_9405873	11/22/21
EPI_ISL_9405885	11/23/21
EPI_ISL_9405878	11/22/21
EPI_ISL_9405884	11/23/21
EPI_ISL_9405889	11/23/21
EPI_ISL_9405887	11/22/21
EPI_ISL_9405888	11/22/21
EPI_ISL_9405890	11/23/21
EPI_ISL_9405894	11/23/21
EPI_ISL_9405899	11/24/21
EPI_ISL_9405904	11/24/21
EPI_ISL_9405902	11/24/21
EPI_ISL_9405905	11/25/21
EPI_ISL_9405898	11/23/21
EPI_ISL_9405897	11/22/21
EPI_ISL_9405896	11/24/21
EPI_ISL_9405903	11/24/21
EPI_ISL_9405901	11/25/21
EPI_ISL_9405900	11/23/21
EPI_ISL_9405908	12/2/21
EPI_ISL_9405907	11/18/21
EPI_ISL_9405906	11/26/21
EPI_ISL_9413625	10/28/21
EPI_ISL_9413639	10/26/21
EPI_ISL_9413645	2/11/21
EPI_ISL_9413657	5/11/21
EPI_ISL_9413659	10/29/21
EPI_ISL_9413669	1/11/21
EPI_ISL_9413671	10/28/21
EPI_ISL_9413623	3/11/21
EPI_ISL_9413648	2/11/21

**Accession ID**	**Sample Collection Date**
EPI_ISL_9413641	1/11/21
EPI_ISL_9413643	1/11/21
EPI_ISL_9413634	1/11/21
EPI_ISL_9413627	10/30/21
EPI_ISL_9413628	10/31/21
EPI_ISL_9413631	10/29/21
EPI_ISL_9413621	3/11/21
EPI_ISL_9413613	1/11/21
EPI_ISL_9413609	10/27/21
EPI_ISL_9413612	10/31/21
EPI_ISL_9413670	10/28/21
EPI_ISL_9413660	2/11/21
EPI_ISL_9413662	10/30/21
EPI_ISL_9413654	3/11/21
EPI_ISL_9413652	3/11/21
EPI_ISL_5687427	8/23/21
EPI_ISL_5687696	7/2/21
EPI_ISL_5687945	7/4/21
EPI_ISL_5688222	7/5/21
EPI_ISL_5689590	6/7/21
EPI_ISL_5689672	6/7/21
EPI_ISL_5689763	7/12/21
EPI_ISL_5689766	7/13/21
EPI_ISL_5689767	7/27/21
EPI_ISL_5689768	7/30/21
EPI_ISL_5689772	8/16/21
EPI_ISL_5689774	8/18/21
EPI_ISL_9413642	3/11/21
EPI_ISL_9413638	1/11/21
EPI_ISL_9413647	1/11/21
EPI_ISL_9413644	10/28/21
EPI_ISL_9413646	4/11/21
EPI_ISL_9413640	10/25/21
EPI_ISL_9413629	10/29/21
EPI_ISL_9413618	2/11/21
EPI_ISL_9413635	10/29/21
EPI_ISL_9413630	10/31/21
EPI_ISL_9413636	2/11/21
EPI_ISL_9413637	1/11/21

Accession ID	Sample Collection Date
EPI_ISL_9413633	1/11/21
EPI_ISL_9413616	10/29/21
EPI_ISL_9413632	10/26/21
EPI_ISL_9413617	2/11/21
EPI_ISL_9413619	10/29/21
EPI_ISL_9413624	10/31/21
EPI_ISL_9413626	10/29/21
EPI_ISL_9413620	3/11/21
EPI_ISL_9413622	4/11/21
EPI_ISL_9413615	2/11/21
EPI_ISL_9413614	2/11/21
EPI_ISL_9413610	10/27/21
EPI_ISL_9413611	10/29/21
EPI_ISL_9413663	4/11/21
EPI_ISL_9413668	10/27/21
EPI_ISL_9413672	10/26/21
EPI_ISL_9413665	5/11/21
EPI_ISL_9413667	3/11/21
EPI_ISL_9413664	10/27/21
EPI_ISL_9413666	1/11/21
EPI_ISL_9413661	4/11/21
EPI_ISL_9413653	2/11/21
EPI_ISL_9413649	3/11/21
EPI_ISL_9413656	5/11/21
EPI_ISL_9413658	3/11/21
EPI_ISL_9413655	5/11/21
EPI_ISL_9413650	3/11/21
EPI_ISL_9413651	1/11/21
EPI_ISL_3188569	5/12/21
EPI_ISL_3188597	5/3/21
EPI_ISL_3188601	5/10/21
EPI_ISL_3188602	5/10/21
EPI_ISL_3188608	5/12/21
EPI_ISL_3188609	5/18/21
EPI_ISL_3188610	5/18/21
EPI_ISL_3188611	5/18/21
EPI_ISL_9405895	11/23/21
EPI_ISL_9405893	11/23/21
EPI_ISL_9405892	11/23/21

**Accession ID**	**Sample Collection Date**
EPI_ISL_9405891	11/24/21
EPI_ISL_9405877	11/22/21
EPI_ISL_9405876	11/23/21
EPI_ISL_9405882	11/23/21
EPI_ISL_9405872	11/22/21
EPI_ISL_9405871	11/22/21
EPI_ISL_9405870	11/22/21
EPI_ISL_9405886	11/23/21
EPI_ISL_9405883	11/23/21
EPI_ISL_9405881	11/23/21
EPI_ISL_9405880	11/22/21
EPI_ISL_9405867	11/22/21
EPI_ISL_9405862	11/17/21
EPI_ISL_9405863	11/18/21
EPI_ISL_9405859	11/16/21
EPI_ISL_9405854	11/15/21
EPI_ISL_9405850	11/15/21
EPI_ISL_9405868	11/22/21
EPI_ISL_9405866	11/18/21
EPI_ISL_9405861	11/15/21
EPI_ISL_9405835	11/10/21
EPI_ISL_9405834	11/10/21
EPI_ISL_9405833	11/12/21
EPI_ISL_9405832	11/11/21
EPI_ISL_9405830	11/12/21
EPI_ISL_9405829	11/3/21
EPI_ISL_9405844	11/15/21
EPI_ISL_9405843	11/12/21
EPI_ISL_9405841	11/11/21
EPI_ISL_9405840	11/12/21
EPI_ISL_3045384	5/21/21
EPI_ISL_3045386	5/21/21
EPI_ISL_3045387	5/21/21
EPI_ISL_3045393	5/21/21
EPI_ISL_3045394	5/21/21
EPI_ISL_3045396	5/18/21
EPI_ISL_3045397	5/21/21
EPI_ISL_3045399	5/20/21
EPI_ISL_3045400	5/20/21

**Accession ID**	**Sample Collection Date**
EPI_ISL_3045403	5/21/21
EPI_ISL_3045404	5/21/21
EPI_ISL_3045414	5/21/21
EPI_ISL_3045415	5/21/21
EPI_ISL_3045420	5/20/21
EPI_ISL_3045422	5/24/21
EPI_ISL_5587623	6/21/21
EPI_ISL_3190269	5/21/21
EPI_ISL_3190270	5/31/21
EPI_ISL_2274000	3/27/21
EPI_ISL_2274004	3/11/21
EPI_ISL_2274025	3/18/21
EPI_ISL_2274026	3/18/21
EPI_ISL_1378834	3/11/21
EPI_ISL_1378835	3/11/21
EPI_ISL_1378836	3/11/21
EPI_ISL_1378837	3/11/21
EPI_ISL_1378838	3/11/21
EPI_ISL_1378839	3/11/21
EPI_ISL_1378840	3/8/21
EPI_ISL_1378841	3/11/21
EPI_ISL_1378842	3/12/21
EPI_ISL_1378844	11/18/20
EPI_ISL_5620856	6/23/21
EPI_ISL_2601034	6/10/21
EPI_ISL_5587680	9/16/21
EPI_ISL_5621287	6/28/21
EPI_ISL_5587611	6/18/21
EPI_ISL_5587617	6/18/21
EPI_ISL_2601037	6/10/21
EPI_ISL_2601038	6/10/21
EPI_ISL_2601035	6/10/21
EPI_ISL_8642669	12/7/21
EPI_ISL_8642603	12/2/21
EPI_ISL_8642634	12/4/21
EPI_ISL_8642625	12/3/21
EPI_ISL_8642630	12/3/21
EPI_ISL_8642627	12/3/21
EPI_ISL_8642632	12/3/21

**Accession ID**	**Sample Collection Date**
EPI_ISL_8642631	12/3/21
EPI_ISL_8642636	12/6/21
EPI_ISL_8642658	12/7/21
EPI_ISL_8642657	12/7/21
EPI_ISL_8642702	12/9/21
EPI_ISL_8642709	12/10/21
EPI_ISL_8642707	12/10/21
EPI_ISL_8642706	12/10/21
EPI_ISL_8642711	12/10/21
EPI_ISL_8642579	11/29/21
EPI_ISL_8642580	11/29/21
EPI_ISL_8642600	12/2/21
EPI_ISL_8642624	12/3/21
EPI_ISL_8642611	12/2/21
EPI_ISL_8642621	12/2/21
EPI_ISL_8642622	12/3/21
EPI_ISL_8642587	12/1/21
EPI_ISL_8642576	11/29/21
EPI_ISL_8642577	11/29/21
EPI_ISL_8642574	11/25/21
EPI_ISL_8642575	11/29/21
EPI_ISL_8642572	11/19/21
EPI_ISL_8642578	11/29/21
EPI_ISL_8642573	11/22/21
EPI_ISL_8642571	11/17/21
EPI_ISL_8642586	11/30/21
EPI_ISL_8642598	12/2/21
EPI_ISL_8642588	12/1/21
EPI_ISL_8642589	12/1/21
EPI_ISL_8642583	11/30/21
EPI_ISL_8642582	11/30/21
EPI_ISL_8642585	11/30/21
EPI_ISL_8642596	12/1/21
EPI_ISL_8642591	12/1/21
EPI_ISL_8642594	12/1/21
EPI_ISL_8642584	11/30/21
EPI_ISL_8642581	11/30/21
EPI_ISL_8642595	12/1/21
EPI_ISL_8642612	12/2/21

**Accession ID**	**Sample Collection Date**
EPI_ISL_8642599	12/2/21
EPI_ISL_8642597	12/2/21
EPI_ISL_8642590	12/1/21
EPI_ISL_8642593	12/1/21
EPI_ISL_8642615	12/2/21
EPI_ISL_8642592	12/1/21
EPI_ISL_8642601	12/2/21
EPI_ISL_8642610	12/2/21
EPI_ISL_8642614	12/2/21
EPI_ISL_8642602	12/2/21
EPI_ISL_8642613	12/2/21
EPI_ISL_8642606	12/2/21
EPI_ISL_8642604	12/2/21
EPI_ISL_8642607	12/2/21
EPI_ISL_8642609	12/2/21
EPI_ISL_8642617	12/2/21
EPI_ISL_8642616	12/2/21
EPI_ISL_8642605	12/2/21
EPI_ISL_8642608	12/2/21
EPI_ISL_8642620	12/2/21
EPI_ISL_8642618	12/2/21
EPI_ISL_8642619	12/2/21
EPI_ISL_8642655	12/6/21
EPI_ISL_8642662	12/7/21
EPI_ISL_8642678	12/8/21
EPI_ISL_8642704	12/9/21
EPI_ISL_8642633	12/3/21
EPI_ISL_8642628	12/3/21
EPI_ISL_8642640	12/6/21
EPI_ISL_8642644	12/6/21
EPI_ISL_8642643	12/6/21
EPI_ISL_8642642	12/6/21
EPI_ISL_8642641	12/6/21
EPI_ISL_8642647	12/6/21
EPI_ISL_8642639	12/6/21
EPI_ISL_8642646	12/6/21
EPI_ISL_8642645	12/6/21
EPI_ISL_8642638	12/6/21
EPI_ISL_8642649	12/6/21

Accession ID	Sample Collection Date
EPI_ISL_8642637	12/6/21
EPI_ISL_8642653	12/6/21
EPI_ISL_8642654	12/6/21
EPI_ISL_8642656	12/6/21
EPI_ISL_8642664	12/7/21
EPI_ISL_8642666	12/7/21
EPI_ISL_8642651	12/6/21
EPI_ISL_8642652	12/6/21
EPI_ISL_8642650	12/6/21
EPI_ISL_8642648	12/6/21
EPI_ISL_8642665	12/7/21
EPI_ISL_8642663	12/7/21
EPI_ISL_8642673	12/7/21
EPI_ISL_8642674	12/7/21
EPI_ISL_8642668	12/7/21
EPI_ISL_8642675	12/7/21
EPI_ISL_8642676	12/8/21
EPI_ISL_8642679	12/8/21
EPI_ISL_8642667	12/7/21
EPI_ISL_8642660	12/7/21
EPI_ISL_8642661	12/7/21
EPI_ISL_8642659	12/7/21
EPI_ISL_8642677	12/8/21
EPI_ISL_8642672	12/7/21
EPI_ISL_8642683	12/8/21
EPI_ISL_8642690	12/8/21
EPI_ISL_8642671	12/7/21
EPI_ISL_8642687	12/8/21
EPI_ISL_8642685	12/8/21
EPI_ISL_8642700	12/9/21
EPI_ISL_8642670	12/7/21
EPI_ISL_8642684	12/8/21
EPI_ISL_8642697	12/8/21
EPI_ISL_8642686	12/8/21
EPI_ISL_8642688	12/8/21
EPI_ISL_8642689	12/8/21
EPI_ISL_8642682	12/8/21
EPI_ISL_8642680	12/8/21
EPI_ISL_8642681	12/8/21

**Accession ID**	**Sample Collection Date**
EPI_ISL_8642698	12/9/21
EPI_ISL_8642695	12/8/21
EPI_ISL_8642699	12/9/21
EPI_ISL_8642693	12/8/21
EPI_ISL_8642694	12/8/21
EPI_ISL_8642696	12/8/21
EPI_ISL_8642701	12/9/21
EPI_ISL_8642712	12/10/21
EPI_ISL_8642691	12/8/21
EPI_ISL_8642705	12/9/21
EPI_ISL_8642714	12/13/21
EPI_ISL_8642626	12/3/21
EPI_ISL_8642716	12/13/21
EPI_ISL_8642713	12/13/21
EPI_ISL_8642703	12/9/21
EPI_ISL_8642708	12/10/21
EPI_ISL_8642635	12/6/21
EPI_ISL_8642710	12/10/21
EPI_ISL_8642715	12/13/21
EPI_ISL_8642623	12/3/21
EPI_ISL_8642629	12/3/21
EPI_ISL_1378833	2/12/21
EPI_ISL_9591620	9/8/21
EPI_ISL_9591264	8/31/21
EPI_ISL_9631583	8/8/20
EPI_ISL_9631585	8/8/20
EPI_ISL_9631714	9/17/20
EPI_ISL_9631639	9/14/20
EPI_ISL_9631619	10/9/20
EPI_ISL_9631584	8/8/20
EPI_ISL_9631610	10/9/20
EPI_ISL_9631644	9/14/20
EPI_ISL_9631621	10/9/20
EPI_ISL_9631645	9/15/20
EPI_ISL_9631623	10/10/20
EPI_ISL_9631795	10/5/20
EPI_ISL_9631804	10/5/20
EPI_ISL_9655614	10/6/20
EPI_ISL_9655775	10/9/20

**Accession ID**	**Sample Collection Date**
EPI_ISL_9631647	9/17/20
EPI_ISL_9631622	10/10/20
EPI_ISL_9655932	10/20/20
EPI_ISL_9655913	10/12/20
EPI_ISL_9662644	10/20/20
EPI_ISL_9662179	10/20/20
EPI_ISL_523811	3/1/20
EPI_ISL_523812	3/4/20
EPI_ISL_525467	5/5/20
EPI_ISL_525469	5/6/20
EPI_ISL_525470	5/6/20
EPI_ISL_525471	5/6/20
EPI_ISL_9665221	11/12/20
EPI_ISL_9668421	11/16/20
EPI_ISL_9670834	11/16/20
EPI_ISL_9670835	11/16/20
EPI_ISL_9670836	11/16/20
EPI_ISL_9670837	11/16/20
EPI_ISL_9670838	11/18/20
EPI_ISL_9670839	11/19/20
EPI_ISL_9670840	11/20/20
EPI_ISL_9670841	11/24/20
EPI_ISL_9670842	11/24/20
EPI_ISL_9670843	11/24/20
EPI_ISL_9670887	11/24/20
EPI_ISL_9670888	11/24/20
EPI_ISL_9670889	11/25/20
EPI_ISL_9671400	11/25/20
EPI_ISL_9671426	11/26/20
EPI_ISL_9671427	11/26/20
EPI_ISL_9671428	11/26/20
EPI_ISL_9671429	11/27/20
EPI_ISL_9671756	12/1/20
EPI_ISL_9671797	12/1/20
EPI_ISL_9671798	12/22/20
EPI_ISL_9671799	1/26/21
EPI_ISL_9671800	1/29/21
EPI_ISL_9671801	1/29/21
EPI_ISL_9671802	1/29/21

**Accession ID**	**Sample Collection Date**
EPI_ISL_9671803	1/29/21
EPI_ISL_9671804	1/29/21
EPI_ISL_9671805	1/29/21
EPI_ISL_9671841	1/29/21
EPI_ISL_9671865	2/3/21
EPI_ISL_9671866	2/5/21
EPI_ISL_9671867	2/8/21
EPI_ISL_9671868	2/15/21
EPI_ISL_9671869	2/15/21
EPI_ISL_9671870	2/16/21
EPI_ISL_9671872	2/16/21
EPI_ISL_9671873	2/16/21
EPI_ISL_9671874	2/23/21
EPI_ISL_9671875	2/25/21
EPI_ISL_9671876	2/16/21
EPI_ISL_9708671	10/14/21
EPI_ISL_9708672	10/14/21
EPI_ISL_9708673	10/15/21
EPI_ISL_9708674	10/15/21
EPI_ISL_9708675	10/18/21
EPI_ISL_9708676	10/18/21
EPI_ISL_9708677	10/18/21
EPI_ISL_9799169	1/25/22
EPI_ISL_9802238	11/30/21
EPI_ISL_9802239	12/2/21
EPI_ISL_9802240	12/13/21
EPI_ISL_9802241	12/2/21
EPI_ISL_9802242	12/13/21
EPI_ISL_9802243	12/2/21
EPI_ISL_9802244	12/13/21
EPI_ISL_9802245	12/15/21
EPI_ISL_9802246	12/6/21
EPI_ISL_9802247	12/20/21
EPI_ISL_9802248	12/20/21
EPI_ISL_9802249	12/20/21
EPI_ISL_9802250	12/20/21
EPI_ISL_9802251	12/27/21
EPI_ISL_9802252	12/27/21
EPI_ISL_9802253	12/21/21

**Accession ID**	**Sample Collection Date**
EPI_ISL_9802254	12/21/21
EPI_ISL_9802255	12/27/21
EPI_ISL_9802256	12/27/21
EPI_ISL_9802257	12/27/21
EPI_ISL_9802258	12/27/21
EPI_ISL_9802259	12/27/21
EPI_ISL_9802260	12/27/21
EPI_ISL_9802261	12/23/21
EPI_ISL_9802262	12/27/21
EPI_ISL_9802263	12/27/21
EPI_ISL_9802264	12/23/21
EPI_ISL_9802265	12/23/21
EPI_ISL_9802266	12/27/21
EPI_ISL_9802267	12/27/21
EPI_ISL_9802268	12/27/21
EPI_ISL_9802269	12/27/21
EPI_ISL_9802270	12/27/21
EPI_ISL_9802271	12/27/21
EPI_ISL_9802272	12/27/21
EPI_ISL_9802273	12/27/21
EPI_ISL_9802274	12/27/21
EPI_ISL_9802275	12/27/21
EPI_ISL_9802276	12/27/21
EPI_ISL_9802277	12/23/21
EPI_ISL_9802278	12/23/21
EPI_ISL_9802279	12/27/21
EPI_ISL_9802280	12/27/21
EPI_ISL_9802281	12/27/21
EPI_ISL_9802282	12/27/21
EPI_ISL_9802283	12/28/21
EPI_ISL_9802284	12/28/21
EPI_ISL_9802285	12/27/21
EPI_ISL_9802286	12/27/21
EPI_ISL_9802287	12/27/21
EPI_ISL_9802288	12/28/21
EPI_ISL_9802289	12/28/21
EPI_ISL_9802290	12/27/21
EPI_ISL_9802291	12/28/21
EPI_ISL_9802292	12/27/21

Accession ID	Sample Collection Date
EPI_ISL_9802293	12/28/21
EPI_ISL_9802294	12/28/21
EPI_ISL_9802295	12/28/21
EPI_ISL_9802296	12/29/21
EPI_ISL_9802297	12/29/21
EPI_ISL_9802298	12/29/21
EPI_ISL_9802299	12/29/21
EPI_ISL_9802300	12/29/21
EPI_ISL_9802301	12/29/21
EPI_ISL_9802302	12/28/21
EPI_ISL_9802303	12/29/21
EPI_ISL_9802304	12/28/21
EPI_ISL_9802305	12/28/21
EPI_ISL_9802306	12/28/21
EPI_ISL_9802307	12/28/21
EPI_ISL_9802308	12/28/21
EPI_ISL_9802309	12/28/21
EPI_ISL_9802310	12/29/21
EPI_ISL_9882176	1/22/22
EPI_ISL_9882177	2/2/22
EPI_ISL_9882178	2/2/22
EPI_ISL_9882179	2/2/22
EPI_ISL_9882180	2/2/22
EPI_ISL_9882181	2/2/22
